# Effects of Hyperhomocysteinemia on the Platelet-Driven Contraction of Blood Clots

**DOI:** 10.3390/metabo11060354

**Published:** 2021-06-01

**Authors:** Rustem I. Litvinov, Alina D. Peshkova, Giang Le Minh, Nail N. Khaertdinov, Natalia G. Evtugina, Guzel F. Sitdikova, John W. Weisel

**Affiliations:** 1 Department of Cell and Developmental Biology, University of Pennsylvania School of Medicine, Philadelphia, PA 19104, USA; litvinov@pennmedicine.upenn.edu; 2 Institute of Fundamental Medicine and Biology, Kazan Federal University, 420008 Kazan, Russia; alinapeshkova26@gmail.com (A.D.P.); mrgiangleminh@gmail.com (G.L.M.); khaertdinofnn@gmail.com (N.N.K.); natalja.evtugyna@gmail.com (N.G.E.); sitdikovaguzel@gmail.com (G.F.S.)

**Keywords:** homocysteine, hyperhomocysteinemia, platelets, blood clotting, contraction of blood clots, retraction of blood clots

## Abstract

Hyperhomocysteinemia (HHcy) is associated with thrombosis, but the mechanistic links between them are not understood. We studied effects of homocysteine (Hcy) on clot contraction in vitro and in a rat model of HHcy. Incubation of blood with exogenous Hcy for 1 min enhanced clot contraction, while 15-min incubation led to a dose-dependent suppression of contraction. These effects were likely due to direct Hcy-induced platelet activation followed by exhaustion, as revealed by an increase in fibrinogen-binding capacity and P-selectin expression determined by flow cytometry. In the blood of rats with HHcy, clot contraction was enhanced at moderately elevated Hcy levels (10–50 μM), while at higher Hcy levels (>50 μM), the onset of clot contraction was delayed. HHcy was associated with thrombocytosis combined with a reduced erythrocyte count and hypofibrinogenemia. These data suggest that in HHcy, platelets get activated directly and indirectly, leading to enhanced clot contraction that is facilitated by the reduced content and resilience of fibrin and erythrocytes in the clot. The excessive platelet activation can lead to exhaustion and impaired contractility, which makes clots larger and more obstructive. In conclusion, HHcy modulates blood clot contraction, which may comprise an underappreciated pro- or antithrombotic mechanism.

## 1. Introduction

Homocysteine (Hcy) is a normal metabolic intermediate produced by demethylation of methionine, an essential dietary amino acid metabolized in the liver and kidneys. Hcy may undergo re-methylation back to methionine or get converted to cystathionine, which is further cleaved to α-ketobutyrate and cysteine. These two metabolic pathways are controlled by S-adenosylmethionine, which is an allosteric inhibitor of methylenetetrahydrofolate reductase that catalyzes the re-methylation of Hcy to methionine (depending on folate and B12 or betaine), while acting as an activator of cystathionine-β-synthase that forms cystathionine (requiring B6) [1]. Deficiencies of the vitamin-containing cofactors, high methionine consumption, genetic or acquired defects in one of the enzymes involved in the Hcy metabolism all lead to elevated levels of plasma Hcy, known as hyperhomocysteinemia (HHcy) [2,3]. In humans, normal levels of plasma Hcy in fasting are 5–12 μM, moderate HHcy is characterized by 12–50 μM, and the high levels for severe HHcy are 50–500 μM [4]. HHcy is present in 5–10% of the general population and the grades of severity are arbitrary and variable [5].

When accumulated in tissues and in the blood above normal levels, Hcy produces toxic effects; therefore, irrespective of the underlying causes and mechanisms, HHcy has a number of clinical implications [6,7]. Many clinical and epidemiological studies have shown that HHcy is associated with increased risk of myocardial infarction, ischemic stroke, and other cardiovascular diseases [8,9,10]. Even mildly elevated blood Hcy increases a risk for cardiovascular disease and thrombotic events, including arterial and venous thrombosis [11,12,13].

To establish the link between HHcy and thrombosis, several studies have been carried out using in vitro addition of exogenous Hcy, ex vivo blood tests in HHcy patients, and animal models of HHcy [14,15]. These and other studies have revealed multiple mechanisms by which Hcy can promote thrombosis, such as platelet activation, hypercoagulability, endothelial dysfunction, and oxidative stress, which leads to up-regulation of tissue factor [16,17,18,19]. Excessive Hcy was found to activate factor V [20], downregulate protein C of the anticoagulant pathway and thrombomodulin expression [21,22,23]. In addition, HHcy was shown to affect the bioavailability of nitric oxide, an inhibitor of endothelial and platelet activation, thus potentiating platelet response to physiological stimulants [24,25]. Despite some progress in elucidating the thrombogenic properties of Hcy, the exact pathophysiologic mechanisms underlying the prothrombotic state of HHcy are not fully understood.

One of the least studied reactions in blood clotting and thrombosis is the volumetric shrinkage of blood clots, which is known as clot contraction or retraction. This process is driven by platelet contractile proteins that generate a mechanical contraction force, pulling on fibrin to compact the clot [26]. Recently, a reduced extent and rate of clot contraction has been identified in the blood of venous thromboembolism patients as well as in patients with ischemic stroke and other (pro)thrombotic conditions, and the reduced ability of clots to contract correlates with the likelihood of thrombosis [27,28]. These findings suggest that pathological changes in the molecular and cellular blood composition that predispose to thrombosis are critical determinants of the lesser ability of clots to contract. Therefore, we hypothesized that Hcy can directly or indirectly affect the kinetics of blood clot contraction and this may be an additional mechanism for association of HHcy with hemostatic disorders. To test this hypothesis, we studied the effects of Hcy on blood clot contraction both in vitro and in vivo using a rat model of HHcy. The results obtained show that Hcy modulates the extent and rate of blood clot contraction and this influence may comprise a novel pro- or antithrombotic mechanism in HHcy.

## 2. Results

### 2.1. Hcy Modulates Clot Contraction In Vitro via Affecting Platelet Functionality

To determine direct effects of Hcy on clot contraction, we incubated normal human and rat blood with addition of exogenous Hcy at various concentrations (5, 20, and 50 μM) and incubation times (1 and 15 min) followed by formation of a clot and monitoring the kinetics of blood clot contraction. L-homocysteine (Santa Cruz Biotechnology, Dallas, TX, USA) was dissolved in saline and added to the blood samples in parallel with a control sample, to which the corresponding amount of solvent without Hcy was added.

**Figure 1:**
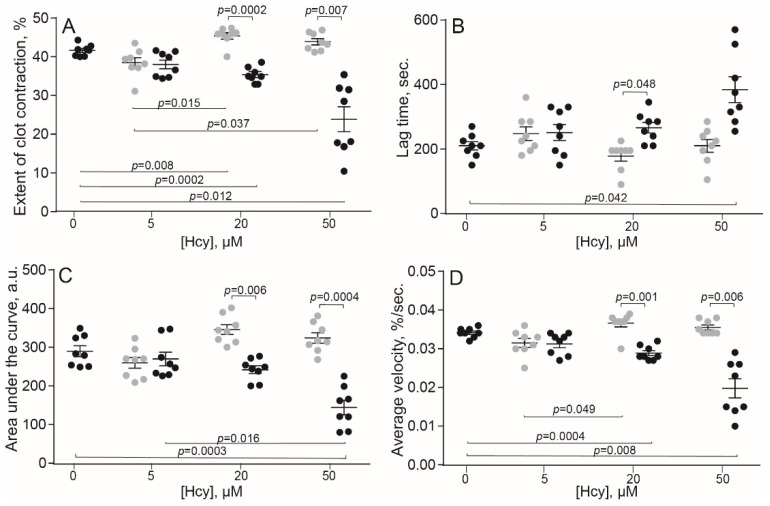
Parameters of clot contraction in in vitro clots made from normal human blood without or with addition of exogenous Hcy in vitro (*n* = 9). Grey dots: 1-min pre-incubation with Hcy; black dots: 15-min pre-incubation with Hcy before the clot contraction assay. Parameters analyzed: extent of contraction (**A**); lag time (**B**); area under the contraction kinetic curve—work done by platelets (**C**); average contraction velocity (**D**). Mean ± SEM, repeated measures ANOVA, post-hoc Tukey’s test. Only significant differences are shown (*p* < 0.05).

**Figure 2:**
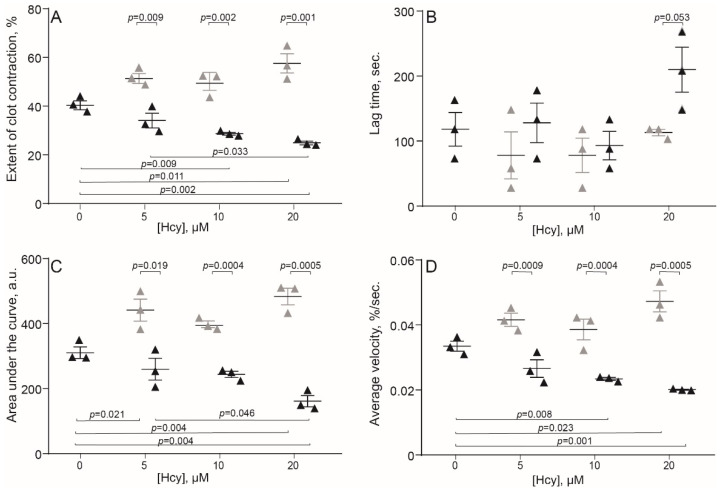
Parameters of clot contraction in clots made from the blood of control rats without and with addition of exogenous Hcy in vitro (*n* = 3). Grey triangles: 1-min pre-incubation with Hcy; black triangles: 15-min pre-incubation with Hcy before initiation of the clot contraction assay. Parameters analyzed: extent of contraction (**A**); lag time (**B**); area under the contraction kinetic curve—work done by platelets (**C**); average contraction velocity (**D**). Mean ± SEM, repeated measures ANOVA, post-hoc Tukey’s test. Only significant differences are shown (*p* < 0.05).

The original levels of Hcy varied from 5 to 10 μM in the human blood and from 3.6 to 7.8 μM in the rat blood, which corresponds to physiological values of 10–12 month old Wistar rats [29]. Compared to the control human blood samples without addition of Hcy, incubation with exogenous Hcy for 1 min increased significantly the extent of clot contraction only at 20 μM Hcy (Figure 1A), indicating that Hcy can enhance clot contraction, but in most cases had no detectable activating effect on top of added thrombin. In contrast, prolonged incubation of blood samples with Hcy for 15 min had the opposite effect and decreased the extent of clot contraction, average velocity and area under the kinetic curve, while increasing the lag-time in a dose-dependent manner (Figure 1). Quite similar dose-dependent effects were observed after 1- and 15-min incubation of the rat blood with Hcy (Figure 2), implying that the influence of Hcy on clot contraction was not species-specific.

**Figure 3:**
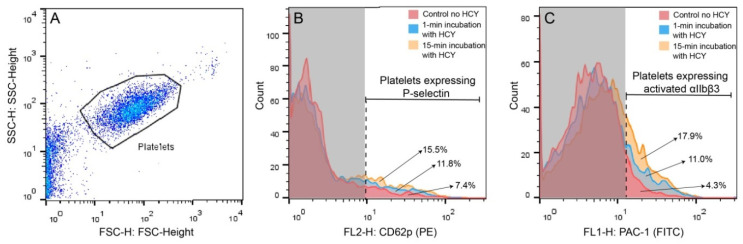
Representative raw data on the flow cytometry of isolated platelets untreated and treated with Hcy. (**A**) Platelets were gated (black closed line) based on the size and granularity using the Forward Light Scatter (FSC) and Side Scatter (SSC) channels, respectively. (**B**,**C**) Platelets were labeled with anti-human CD62 P phycoerythrin-conjugated antibodies (**B**) or fluorescein isothiocyanate-conjugated PAC-1 antibodies (**C**) before and after addition of 50 μM Hcy followed by 1-min or 15-min incubation. B and C represent combined histograms showing the fractions (%) of the fluorescent platelets under different experimental conditions. The vertical dashed line separates the non-fluorescent (on the left) and fluorescent signals within the platelet gate. Each histogram contains 5000 total counts.

A conceivable explanation of the results obtained is that Hcy can directly activate platelets, promoting their contractility, followed by time-dependent platelet exhaustion and suppression of the ATP-dependent contraction, as shown previously for other platelet stimulants and pathological conditions [27,30,31,32]. To test this assumption and reveal the true Hcy-induced platelet activation, we analyzed effects of Hcy on platelet functionality in the absence of thrombin or any other platelet stimulants. Human platelet-rich plasma (PRP) was treated with 50 μM Hcy for 1 or 15 min and then flow cytometry of platelets was used to assess the surface expression of P-selectin and active integrin αIIbβ3, the molecular markers of platelet activation. Figure 3 shows representative raw data on the flow cytometry of isolated platelets untreated and treated with Hcy. The average results summarized in Table 1 show that treatment with Hcy induced substantial time-dependent platelet activation compared to the control Hcy-untreated platelets. After treatment of resting platelets with Hcy for 15 min, the average fraction of platelets expressing P-selectin was increased two-fold and expression of the active integrin αIIbβ3 was increased ~five-fold compared to control. There was also a significant difference between the average values for 1- and 15-min incubation. These data strongly suggest that the time-dependent effects of Hcy on clot contraction in vitro are due to the Hcy-induced platelet activation, including their enhanced contractility, associated with the gradual energetic exhaustion and dysfunction of platelets, manifesting later as impaired clot contraction.

### 2.2. HHcy Modulates Clot Contraction In Vivo

**Figure 4:**
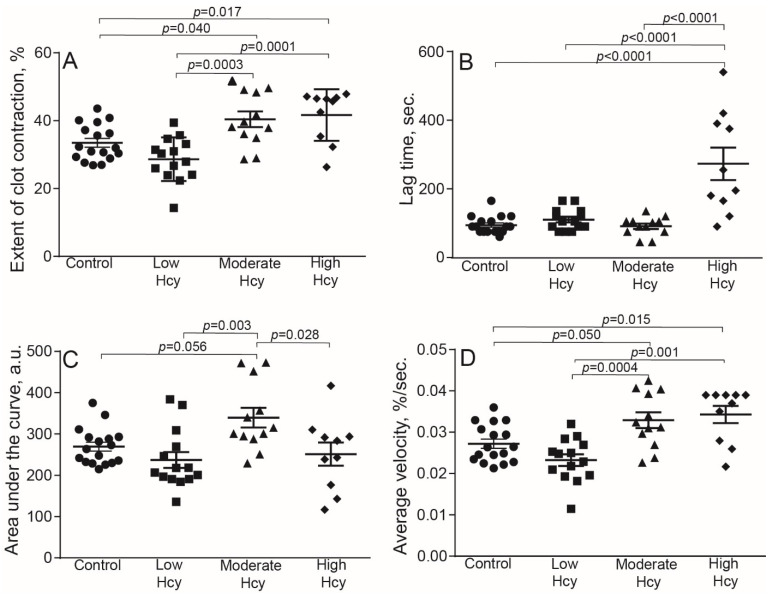
Parameters of blood clot contraction in clots made from the blood of control and HHcy rats as a function of Hcy levels in plasma: normal control (3.5–7.8 μM, *n* = 17), low in methionine-resistant rats (3.6–9.9 μM, *n* = 14), moderately elevated (10–50 μM, *n* = 12) and high (51–122 μM, *n* = 10). Parameters analyzed: extent of contraction (**A**); lag time (**B**); area under the contraction kinetic curve—work done by platelets (**C**); average contraction velocity (**D**). Mean ± SEM, ordinary one-way ANOVA, post-hoc Tukey’s test. Only significant differences are shown (*p* < 0.05).

To assess the effect of Hcy on blood clot contraction in physiological conditions, we used a well-established experimental HHcy model in rats with continuously elevated blood Hcy levels [33,34,35,36,37]. In this model methionine load results in an increase of S-adenosylhomocysteine level, which subsequently hydrolyses to homocysteine and adenosine, inducing HHcy [33]. The HHcy rats were divided into three sub-groups based on the Hcy concentrations in plasma: low (3.6–9.9 μM), moderate (10–50 μM), and high (51–122 μM). The results are summarized in Table 2 and Figure 4. The low HHcy rats comprised a subpopulation of animals that were resistant to methionine diet and their Hcy levels and the parameters of clot contraction were indistinguishable from control. In the moderate and high HHcy rats, the extent and average velocity of clot contraction were enhanced significantly compared to the control and low HHcy sub-group (Figure 4A,D). The area under the kinetic curve was higher in the moderate HHcy sub-group compared to all other groups of animals (Figure 4C). On the contrary, the lag time of clot contraction was prolonged in the rats with the highest Hcy levels (50–122 µM), compared to the control and other HHcy sub-groups (Figure 4B). These dual effects of the in vivo HHcy on contraction of blood clots indicate that clot contraction is affected by elevated Hcy levels in blood strongly but oppositely. The lack of sharp dose-dependency may arise because clot contraction is a complex multifactorial process that is modulated in vivo by many uncontrollable variables other than Hcy levels. In addition, Hcy has multiple physiologic effects, which may interfere and be a source of data inconsistency in the HHcy model.

### 2.3. HHcy-Induced Changes in the Blood Composition That Can Affect Clot Contraction

In addition to the effects of HHcy on platelet functionality, clot contraction could be altered due to Hcy-induced changes in blood composition. It has been shown previously that the clot contraction kinetics is affected by platelet count, hematocrit, and fibrinogen levels [38]. All these three parameters were changed substantially in the HHcy rats, depending on the Hcy levels in blood (Table 3). The platelet count was increased significantly in the high HHcy sub-group of animals compared to the control and other HHcy rats. The increase in platelet count was associated with a significant reduction in the red blood cell count and lower fibrinogen levels. In the moderate HHcy rats, the changes in blood composition had the same trend, but only the reduced red blood cell count reached the level of statistical significance.

To confirm a link between blood composition and clot contraction in the HHcy rats, we performed a correlation analysis between the parameters of clot contraction and blood tests (Table 4). There was a strong positive correlation between the Hcy levels and the extent and velocity of clot contraction, supporting the promoting effect of Hcy on contraction over inhibition. The Hcy levels also correlated positively with the contraction lag time, confirming the dose-dependent delay in the onset of clot contraction shown in Figure 4B. Expectedly, both fibrinogen levels and red blood cells counts displayed strong negative correlation with the extent and average velocity of clot contraction; the red blood cell count also correlated negatively with the area under the kinetic curve of clot contraction. Paradoxically, the platelet count showed positive, yet weak, correlation with the lag-time, suggesting indirectly that the delay of clot contraction might be due to a higher absolute number of dysfunctional platelets. Taken together, these data suggest that the HHcy-related enhanced contraction of blood clots can be partially due to the reduced hematocrit and lower amount fibrin(ogen) in the blood of the HHcy rats. These and other potential pathophysiologic effects of HHcy may explain the seeming inconsistency of the effects of Hcy on clot contraction in vitro and in vivo, at least with respect to the time-dependency.

## 3. Discussion

An elevated level of Hcy in blood has been established as a risk factor for cardiovascular disease and thrombosis, but the pathogenic mechanisms of thrombogenicity in HHcy remain controversial and largely unclear. This is partially because many aspects of thrombosis continue to be enigmatic and the triggers and promoting factors of this life-threatening pathologic condition are not fully understood. Recently, there has been a revival of attention to contraction or retraction of blood clots as an underappreciated and understudied process that has a major pathogenic and clinical significance in (pro)thrombotic conditions of various etiologies [27,28,30,31,39,40]. Importantly, the contraction or shrinkage of a blood clot can occur not only in vitro, but also in vivo, and, therefore, can cause intravital structural remodeling of arterial and venous thrombi that may have various pathophysiological consequences [27,30,41,42]. The most apparent sequelae of the compaction of thrombi include changes of the vessel lumen, thereby modulating local blood flow in the thrombotic occlusion area [30]. The extent of thrombus compression and densification can determine the likelihood of its mechanical rupture, i.e., thrombotic embolization [27]. In addition, dense compacted thrombi become impermeable for pathogens and fibrinolytic enzymes [43], as well as acquire altered susceptibility to external and internal fibrinolysis [44]. With all these factors in mind, we sought to investigate if Hcy can change the ability of blood clots to contract as a potential mechanism contributing to thrombogenicity in HHcy.

Both in vitro and in vivo studies have revealed dual effects of Hcy on the kinetics of blood clot contraction that depend on the concentration of Hcy and its time of action. Clot contraction is accelerated and promoted after a short-term incubation of blood with exogenous Hcy and at a moderate and high level of endogenous Hcy in an animal model of HHcy (Figure 1, Figure 2 and Figure 3 and Table 2). The effects turn to the opposite, i.e., suppression of clot contraction, at a prolonged incubation time of blood with Hcy in vitro or at a higher Hcy concentration in the blood of HHcy rats (Figure 1, Figure 2 and Figure 3 and Table 2). This seeming contradiction can be explained based on the complex physiology of platelets, the main driver of blood clot contraction, which can exist in various functional states.

Activated platelets are mechanically active cells that contain the ATP-dependent cytoskeletal machinery necessary for generation of traction forces [45]. These forces are transmitted to fibrin fibers outside of the platelet attached to the extracellular portion of the integrin αIIbβ3. Using a complex biomechanical mechanism, platelets pull on the fibrin fibers, leading to the volumetric shrinkage of a clot, densification of the fibrin network, and formation of fibrin-platelet agglomerates [26]. What turns this contractile machinery on is platelet activation by thrombin and other physiological stimulants. Hcy has been shown to act as a platelet activator both directly and indirectly [13,19,24,25,46,47,48,49,50,51]. By measuring the expression of P-selectin and active integrin αIIbβ3, we demonstrated direct Hcy-induced platelet activation (Table 1), which agrees with the ability of Hcy to trigger tyrosine phosphorylation and phospholipase Cγ2 through generation of reactive oxygen species and TxA_2_ [46]. Expression of adhesive receptors P-selectin and active αIIbβ3 on platelets may be a potent mechanism for an increased thrombotic risk in HHcy. Notably, the platelet-stimulating activity in vivo could be attributed not to Hcy itself, but rather to its super-active thioester metabolic derivative, Hcy thiolactone [52,53]. Exogenous Hcy was shown to potentiate collagen-induced platelet activation through signaling pathways linked to receptor molecules glycoprotein VI and integrin α2β1 [47]. This observation implies a possibility that Hcy or its derivatives can “prime” platelets, making them particularly sensitive to physiological activators, such as thrombin, which is used to trigger platelet contractility in the clot contraction assay. In addition to direct dose-dependent platelet-activating effects, Hcy can activate platelets indirectly via multiple in vivo physiologic mechanisms, such as suppressed formation of nitric oxide (NO), an inhibitor of platelet activation [24,25,48]. Another inhibitor of platelet activation, whose production is reduced in HHcy, is hydrogen sulfide (H_2_S) synthesized from cysteine, a metabolic product of Hcy [49,50,51]. There are other potential mechanisms of platelet activation in HHcy [13,19], although there is still a controversy regarding whether the main stimulating effect of Hcy on platelets in vivo is direct or mediated via its action on other cells [54].

While the Hcy-induced platelet activation, direct and indirect, explains the enhancement of blood clot contraction in the HHcy conditions, the ability of Hcy to suppress clot contraction at higher concentrations and/or after longer incubation with blood suggests that under certain circumstances Hcy can cause platelet dysfunction. In our clinical studies, the impaired contraction of blood clots was observed in a number of (pro)thrombotic conditions and has been attributed to energetic exhaustion following continuous or chronic platelet activation [27,30,31,39,40]. There is a good reason to postulate that similar mechanisms of reduced platelet contractility apply to HHcy: Hcy can progressively activate platelets (Table 1), including triggering ATP-dependent force generation, and then render them partially exhausted, refractory, and unable to undergo full contraction. The potentially contradictory effects of Hcy on platelet contractility revealed here may account for some reports that do not support prothrombotic effects of moderate HHcy, including the lack of Hcy-induced platelet activation or potentiating effects of Hcy on ADP-induced platelet aggregation [55].

The biological and mechanical properties of fibrin is another variable that can affect the ability of a blood clot to shrink in response to platelet-mediated contraction. The impact of Hcy on fibrin is multifarious and can results in alterations of structure and mechanics of the fibrin network that have important clinical implications [56]. The HHcy-induced hypofibrinogenemia (Table 3) could facilitate clot contraction because fibrinogen concentration is inversely related to the extent and rate of clot contraction [38]. Hcy can also cause qualitative changes in fibrin(ogen) in vivo due to the interaction of Hcy with cysteine residues [57] or binding of Hcy thiolactone to the primary amino groups of lysines [58,59]. The Hcy-modified fibrinogen is converted to atypical fibrin clots that have impaired kinetics of fibrin formation [59,60,61], abnormal structure of individual fibrin fibers [62] and arrangement of the entire network [59,63,64]. Such clots have substantially reduced clot permeability and sensitivity to the enzymatic lysis [64,65,66,67]. Importantly, Hcy changes viscoelasticity and stiffness of fibrin [67,68], which can promote or suppress clot contraction, depending on the reduced or increased resilience of the fibrin scaffold, respectively.

All three main constituent parts of clots and thrombi, that is RBCs, platelets, and fibrin, contribute to the process of contraction. While stimulated platelets comprise the active contractile element, fibrin, and RBCs represent mechanically passive structures that resist contraction. As a result of biomechanical and structural remodeling, RBCs are compressed inside a clot or thrombus and acquire the shape of polyhedra; hence, they have been called polyhedrocytes [43]. Given the mechanical resilience of RBCs and their occupancy of space in the clot, there is an expected inverse correlation between hematocrit and the extent of blood clot contraction [38]. Since the HHcy rats had low RBC counts compared to control animals (Table 3), the reduced volume fraction of RBCs in blood clots provides an additional mechanism for the enhanced contraction of clots formed in the blood of the HHcy animals.

Taken together, our results suggest that in HHcy, platelets get activated directly and indirectly, leading to enhanced clot contraction that is also facilitated by the reduced content and resilience of fibrin and erythrocytes in the clot. After Hcy-induced activation, some platelets later become partially dysfunctional and less contractile, resulting in reduced extent and rate of clot contraction. The dual effect of Hcy on blood clot contraction may comprise a novel mechanism of hemostatic disorders associated with HHcy.

## 4. Materials and Methods

### 4.1. Human Blood Samples

Volunteers were excluded from this study if for any reason they took anticoagulants, thrombolytics, or antiplatelet drugs at least 14 days prior to examination. Human blood was obtained from 9 healthy subjects 21–47 years old (average 28 ± 8 years), of which 1 (11%) was a man and 8 (89%) were women, all with BMI < 25. The same blood samples were analyzed for contraction of blood clots and flow cytometry at various concentrations of Hcy and times of incubation.

For experiments with whole blood and platelet-rich plasma (PRP), blood was drawn by venipuncture from healthy volunteers. Informed consent was obtained from all subjects involved in the study in accordance with the Declaration of Helsinki and requirements of the local Ethics Committee of Kazan Federal University (resolution No. 27 as of 28 December 2020). All procedures were carried out in accordance with the approved guidelines. To obtain PRP, whole blood was stabilized with 3.8% sodium citrate (9:1 *v*/*v*) and then centrifuged at 200× *g* for 10 min within ½ hour after blood withdrawal to obtain platelet-rich plasma (PRP). The whole citrated blood and PRP were processed and kept at room temperature and used within 4 h after blood collection.

### 4.2. Blood Clot Contraction Assay

**Figure 5:**
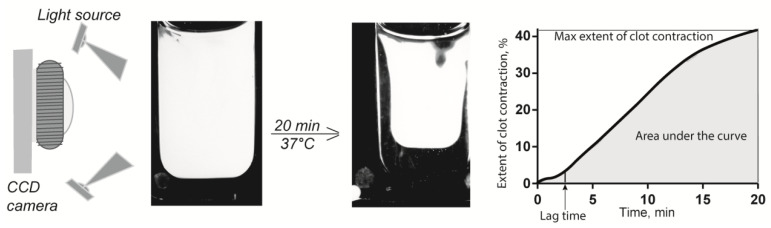
Schematic of the optical system used to quantify clot contraction. The change in clot size is registered automatically by the CCD camera and presented as a kinetic curve that is characterized by numeric parameters.

The kinetics of contraction of blood clots was tracked optically using a Thrombodynamics Analyzer System (Figure 5) (HemaCore Ltd., Moscow, Russia). Citrated blood from humans or rats without or with addition of Hcy was activated with 2 mM CaCl_2_ (final concentration) and 1.5 U/mL human α-thrombin (Sigma-Aldrich, St. Louis, MO, USA) with rat blood or 1.0 U/mL human α-thrombin (Sigma-Aldrich, USA) with human blood. The activated blood samples (80 μL) were quickly transferred to a 12 mm × 7 mm × 1 mm transparent plastic cuvette, which was pre-coated with a thin layer of 4% *v*/*v* Triton X-100 in 150 mM NaCl to prevent the clot from sticking to the walls of the chamber without affecting the clot structure and platelet functionality. The transparent cuvette was placed at 37 °C into the temperature-controlled chamber of the optical analyzer. The experiments were performed in duplicate simultaneously using double-spaced cuvettes. Light scattering-based images of the contracting clots were taken every 15 s for 20 min to track changes in the clot size (Figure 5). The serial images were analyzed computationally to plot a kinetic curve of clot contraction and extract the following parameters: (i) extent of contraction calculated as [(*S*_0_ − *S_t_*)/*S*_0_] × 100, where *S*_0_ is the initial clot size and *S_t_* is the final clot size at the 20-min end point; (ii) lag time determined as the time from the addition of thrombin until the clot reaches 95% of its initial size; (iii) the average contraction velocity, which is an average of the first derivative of the contraction kinetic curve; and (iv) the area under the contraction kinetic curve, corresponding to the amount of mechanical work on clot compression done by the contracting platelets.

### 4.3. Flow Cytometry of Human Platelets

Flow cytometry of platelets was performed in PRP diluted with Tyrode’s buffer to adjust the platelet count to 8 × 10^6^/mL. 400,000 platelets in 50 μL of the diluted human PRP were incubated with Hcy (50 μM final concentration) for 1 or 15 min at 37 °C. Then platelets were labeled for 10 min at 25 °C by double staining with 1 μg/mL fluorescein isothiocyanate-conjugated PAC-1 antibodies against activated human αIIbβ3 (BD Biosciences, San Jose, CA, USA) and 0.045 μg/mL anti-human-CD62P antibodies conjugated with phycoerythrin (BD Biosciences, USA). After incubation with the labeled antibodies, platelets were analyzed using a FacsCalibur flow cytometer equipped with BD Cell-Quest Pro software. Platelets were gated based on their size and granularity and 5000 platelets were analyzed in each sample. FlowJo Version 10 software was used for data analysis.

### 4.4. Rat Model of Hyperhomocysteinemia (HHcy)

Experiments were carried out on Wistar rats in accordance with EU Directive 2010/63/EU for animal experiments and requirements of the Local Ethical committee of Kazan Federal University (resolution No. 8 as of 5 May 2015). All procedures were carried out in accordance with the approved guidelines. Animals were housed in polypropylene cages (32 × 40 × 18 cm) under controlled temperature (22–24 °C) with a 12:12 L/D light schedule (lights on at 6:00 am) and free access to food and water. To induce HHcy, male rats aged from 10 to 12 months received methionine (7.7 g/kg body weight daily) with food for 8–16 weeks [33,34,35,36,37]. Blood from the heart of control and HHcy rats was mixed with 3.8% sodium citrate (9:1 *v*/*v*). Citrated blood and plasma from the rats were used for the clot contraction assay, hemostatic tests, and measuring the Hcy concentrations within 2 h after blood collection.

### 4.5. Determination of Fibrinogen Concentration in Blood Plasma

Whole citrated blood from rats was centrifuged at room temperature for 10 min at 1500× *g* and then for 10 min at 10,000× *g* to obtain platelet-free plasma (PFP). Clottable fibrinogen was converted to fibrin by adding human thrombin (5 U/mL final concentration) to 200 μL of PFP with at 37 °C. After 30 min, the clot was washed with saline (3 times × 30 min) and left overnight to wash plasma proteins. The clot was blotted and dissolved in 400 μL of 0.25 M NaOH by heating in boiling water for 5 min. The absorbance was determined at 280 nm in a Nanodrop Lite spectrophotometer (ThermoFisher Scientific, Waltham, MA, USA) and converted into protein concentration. The specific absorption coefficient for fibrin(ogen) was 1.51 for 1 mg/mL in a 1-cm cuvette and calculations were done using the following formula: *C* = (*A*_280_/1.51) × *T* × *P*), where *C* is fibrin concentration, *A*_280_ is the measured absorbance, *T* is a beam path or width of the cuvette (1 cm), and *P* is correction for the sample dilution. The amount of fibrin was equal to the amount of fibrinogen in the initial plasma sample.

### 4.6. Determination of Hcy Levels in Blood Plasma

Total Hcy in plasma was measured using a nanocarbon modified electrode as previously described [69,70,71]. Briefly, 300 μL of platelet-poor plasma mixed with 40 μL of catechol and 3.66 mL of phosphate buffer (pH 7.0) were added to the electrochemical cell and square-wave voltammograms were recorded within the potential range from 0.5 to −0.4 V using the Potentiostat µAutolab type III with the software GPES (Eco Chemie B.V., Utrecht, The Netherlands). Baseline correction was made using GPES software for better peak identification. Hcy concentration was calculated using a calibration graph. Hcy levels of the control group ranged within 3.5–7.8 μM, while in the HHcy animals the range was 3.6–122 μM; therefore, the HHcy animals were segregated into three subgroups depending on the plasma levels of Hcy: low (3.6–10.0 μM, *n* = 14), moderate (10–50 μM, *n* = 12), and high (50–122 μM, *n* = 10).

In the in vitro experiments, exogenous Hcy at 5 μM, 10 μM, 20 μM, and 50 μM final concentrations was added to human citrated blood samples incubated for 1 or 15 min or rat for 15 min at 37 °C before blood testing.

### 4.7. Statistical Analysis

Statistical analyses were performed using GraphPad Prism 7 (GraphPad Software, La Jolla, CA, USA). Normality of data distribution was assessed with the Shapiro–Wilk and D’Agostino–Pearson criteria. Pairwise statistical differences were estimated using the Student’s *t*-test (parametric analysis) and Mann-Whitney test (nonparametric analysis). Statistical differences for multiple comparisons were estimated using the one-way ANOVA with the post-hoc Tukey’s test. Correlation analysis was performed using the Spearman’s rank correlation coefficient. The level of significance was 95% (*p* < 0.05). In our observations, the effect sizes of all the data were large (>1) and the statistical power was approximately 80% or higher.

## 5. Conclusions

Hcy has been demonstrated to modulate the extent and rate of blood clot contraction and this influence may comprise a novel pro- or antithrombotic mechanism in HHcy. Depending on the Hcy concentration and time of action, Hcy can either enhance or impair the kinetics of clot contraction, but in the blood of HHcy animals, the activating effect of HHcy prevails, at least at moderate Hcy levels (10–50 μM). The stimulating effect of Hcy on blood clot contraction in vivo is due to direct or indirect Hcy-induced platelet activation and thrombocytosis combined with reduced hematocrit and hypofibrinogenemia, leading to reduced content and smaller mechanical resilience of fibrin and RBCs in the clot. An increased expression of adhesive molecules, P-selectin and active integrin αIIbβ3, is a strong thrombogenic mechanism for HHcy. Despite the overall stimulating effect of moderate HHcy on clot contraction, in the blood of rats with the higher Hcy levels (>50 μM), the onset of clot contraction was delayed, suggesting partial refractoriness of platelets, likely resulting from continuous activation and exhaustion. Taken together, the data suggest that blood clot contraction in HHcy is altered, which may have pro- or antithrombotic effect depending on the degree of HHcy. At moderate Hcy levels, despite a higher risk of thrombosis, well-compacted clots and intravascular thrombi can be less obstructive, more prone to internal fibrinolysis, and less embologenic. At higher Hcy levels, the contraction may be partially impaired, resulting in prothrombotic consequences, such as formation of more occlusive thrombi that are more prone to embolization and less sensitive to physiological fibrinolysis. In addition, the results obtained point to a potential diagnostic and prognostic value of the clot contraction assay as a novel test for ongoing or threatening thromboembolism in HHcy.

## Figures and Tables

**Table 1:** Effects of exogenous Hcy (50 µM) on the expression of platelet activation markers.

Markers	Untreated Platelets (*n* = 4)	Hcy-Treated Platelets (*n* = 4)	
		1-min Incubation	15-min Incubation
P-selectin	6.9 (5.3–8.6)	8.4 (5.4–11.3) ^†^	12.9 (11.5–15.8) *
Activated integrin αIIbβ3	2.8 (2.0–3.0)	9.7 (3.5–13.4) ^†^	15.4 (11.7–18.0) *

The numbers represent a fraction of platelets (%) that bind fluorescently labeled antibodies against P-selectin or against activated integrin αIIbβ3 in the flow cytometry assay. Mean (IQR), paired *t*-test. * Statistically significant compared with control. ^†^ Statistically significant compared with the 15 min incubation.

**Table 2:** Clot contraction parameters in clots made from the blood of control and HHcy rats with low, moderate and high Hcy levels.

Parameters	Hcy Levels in Blood			
	Control 3.6–7.8 μM (*n* = 17)	Low HHcy 3.6–9.9 μM (*n* = 14)	Moderate HHcy 10–50 μM (*n* = 12)	High HHcy 51–122 μM (*n* = 10)
Extent of clot contraction, %	33.5 ± 1.3 ^§^	28.6 ± 1.7 ^§^	40.4 ± 2.3 *^,^^†^	41.7 ± 2.4 *^,^^†^
Lag time, s	94 ± 6 ^§^	110 ± 8 ^§^	91 ± 9 ^§^	273 ± 47 *^,^^†^
Area under curve, a.u.	270 ± 11	237 ± 19	340 ± 24 ^†,§^	252 ± 28

Mean ± SEM, ordinary one-way ANOVA, post-hoc Tukey’s test. * Statistically significant compared with control. ^†^ Statistically significant compared with the low HHcy subgroup. ^§^ Statistically significant compared with the high HHcy subgroup.

**Table 3:** Blood composition of control rats compared to the HHcy rats with low, moderate, and high Hcy levels.

Parameters	Hcy Levels in Blood			
	Control 3.6–7.8 μM (*n* = 17)	Low HHcy 3.6–9.9 μM (*n* = 14)	Moderate HHcy 10–50 μM (*n* = 12)	High HHcy 51–122 μM (*n* = 10)
Fibrinogen, g/L	3.5 ± 0.2 ^§^	3.1 ± 0.4 ^§^	2.4 ± 0.4	1.3 ± 0.2 *^,†^
Platelets, ×10^12^/L	1.2 ± 0.1 ^§^	1.3 ± 0.2 ^§^	1.0 ± 0.3 ^§^	13.2 ± 3.1 *^,†^
Red blood cells, ×10^12^/L	5.3 ± 0.4	6.6 ± 0.7 ^§^	3.6 ± 0.3 *^,†^	3.9 ± 0.4 ^†^

Mean ± SEM, ordinary one-way ANOVA, post-hoc Tukey’s test. * Statistically significant compared with control. ^†^ Statistically significant compared with the low HHcy subgroup. ^§^ Statistically significant compared with the high HHcy subgroup.

**Table 4:** Correlation analysis between the parameters of in vitro clot contraction and blood composition in the HHcy rats.

Parameters of Blood Composition	Parameters of Blood Clot Contraction Kinetics			
	Extent of Clot Contraction	Lag Time	Area under Curve	Average Velocity
Hcy blood levels in vivo	0.56 ***	0.43 **	-	0.56 ***
Fibrinogen levels	−0.37 ***	-	-	−0.38 **
Platelet counts	-	0.29 *	-	-
RBCs counts	−0.42 **	-	−0.29 *	−0.42 **

Only significant Spearman’s coefficients are shown: * *p* < 0.05; ** *p* < 0.01; *** *p* < 0.001.

## Data Availability

The data presented in this study are available in the article.
